# Ocular microvascular alteration in patients with myocardial infarction—a new OCTA study

**DOI:** 10.1038/s41598-023-50283-1

**Published:** 2024-02-24

**Authors:** Jun-Yi Wu, Jin-Yu Hu, Qian-Min Ge, San-Hua Xu, Jie Zou, Min Kang, Ping Ying, Hong Wei, Qian Ling, Liang-Qi He, Cheng Chen, Yi Shao

**Affiliations:** 1 Department of Ophthalmology, Eye & ENT Hospital of Fudan University, Shanghai, 200030 China; 2 Department of Ophthalmology, The First Affiliated Hospital, Jiangxi Medical College, Nanchang University, Nanchang, 330006 Jiangxi China

**Keywords:** Cardiology, Diseases

## Abstract

Myocardial infarction is defined as a sudden decrease or interruption in blood flow to the coronary arteries, causing ischemic necrosis of the corresponding cardiomyocytes. It is unclear whether systemic macrovascular alterations are associated with retinal microvascular changes. This study utilized optical coherence tomography angiography (OCTA) to compare variations in conjunctival vascular density and fundus retinal vessel density between patients with myocardial infarction (MI) and healthy controls. This study recruited 16 patients (32 eyes) with MI and 16 healthy controls (32 eyes). The superficial retinal layer (SRL), deep retinal layer (DRL) and conjunctival capillary plexus in each eye were evaluated by OCTA. Parameters measured included the density of the temporal conjunctival capillary, retinal microvascular (MIR) and macrovascular (MAR) alterations and total MIR (TMI). The microvascular density of each retinal region was evaluated by the hemisphere segmentation (SR, SL, IL, and IR), annular partition (C1, C2, C3, C4, C5 and C6), and modified early treatment of diabetic retinopathy study (R, S, L, and I) methods. In the macular area, the superficial and deep retinal microvascular densities displayed notable variations. In the superficial layers, the superficial TMI, superficial MIR, and superficial MAR, as well as densities in the SL, IL, S, L, C1, C2, C5 and C6 regions, were significantly lower in MI patients (p < 0.05 each). In the deep layers, the deep MIR and deep TMI), as well as densities in the SL, IL, L, C1, C2 and C6 regions were significantly lower in MI patients (p < 0.05 each). In contrast, the conjunctival microvascular density was significantly higher in MI patients than in healthy controls (p < 0.001). The microvascular densities measured in the deep and superficial retinal layers and in the conjunctiva differ in MI patients and healthy controls. OCTA is effective in detecting changes in the ocular microcirculation.

## Introduction

Myocardial infarction (MI) is the abrupt ischemic death of myocardial tissue. As a serious cardiovascular disease, MI is a major cause of disability and death in the global population^1^. MI is usually caused by progressive coronary artery atherosclerosis and thrombosis, resulting in an insufficient supply of blood and oxygen to the myocardium, necrosis of cardiomyocytes, and poor cardiac remodeling^2^. Symptoms indicative of MI include chest pain, stomach pain, shortness of breath, nausea, vomiting, and an irregular heartbeat^3^. Factors associated with MI include aging, hypertension, diabetes, high levels of low density lipoprotein (LDL), obesity, heavy drinking, and drug overdose^4^. Cardiac and retinal blood vessels have the same histological origin, similar microcirculatory anatomy and blood vessel level, and are exposed to the same internal environments. The morphological structure and physiological function of retinal blood vessels are closely influenced by systemic vascular diseases^5,6^. Moreover, the retina is a special location that allows direct visualization of its microvasculature^7^.

The standard techniques used to evaluate the posterior route in MI patients include fundus fluorescence angiography (FFA) and indocyanine green angiography (ICGA), methods that have been used to diagnose vascular diseases. FFA and ICGA are invasive fundus angiography techniques, which can reflect fundus blood perfusion and show the size, shape and location of the lesion. Superimposition of the images of superficial and deep fundus microvessels visualized by these techniques, however, do not clearly show the microscopic structure and density of the capillaries present in the entire retina. Some patients may also be allergic to contrast agents or may experience clinical discomfort, such as vomiting and fainting.

Recent studies have used optical coherence tomography angiography (OCTA) to evaluate the connections between heart disease and alterations in the ocular microvasculature^8^. Changes in the characteristics of the retinal microvasculature and variations in the thickness and layering of the retina have been associated with cardiovascular health^9,10^. OCT and OCTA have been found to be important, secure, and noninvasive methods in the detection of coronary artery disease. Reductions in vascular density and in choroidal and retinal thickness in the central retinal region were reported to be predictive of coronary artery disease^11^. OCTA, which does not require injection of contrast media, is a non-invasive technique providing three-dimensional visualization of the perfused vasculature of the retinal vessels^12^. Specifically, OCTA can visualize the continuous movement of red blood cells within blood vessels in the same fundus. By combining high-resolution blood vessel imaging with motion contrast imaging, OCTA can accurately evaluate the blood vessels in the retina and choroid^13^. OCTA automatically divides fundus blood flow images into four layers: superficial retinal capillaries (from the inner border membrane to the inner plexus layer), deep retinal capillaries (from the inner plexus layer to the outer plexus layer), outer retinal capillaries (from the outer plexus layer to the glass membrane) and choroidal capillaries (below the glass membrane)^14^. The blood flow density of each layer can be quantitatively determined by an EnFace model. Dense volume scans can produce OCTA images that are comparable to standard FFA images. In contrast to FFA, however, OCTA offers a distinct advantage, as it does not require dye injections, thus avoiding the adverse reactions associated with contrast media.

In ophthalmology, OCTA can quantitatively analyze the hemodynamics, blood vessel density and blood vessel network morphology in patients with fundus vascular diseases, providing a more accurate and reliable basis for disease diagnosis and treatment. OCTA is also widely used in the diagnosis of systemic diseases. For example, OCTA evaluation of patients with hypertension has shown that poor control of hypertension was associated with focal ischemia of the choroid, which may be caused by choroid endothelial injury and vasoconstriction^15^. OCTA evaluation of patients with chronic kidney disease (CKD) found that retinal vascular density and the circularity index of the foveal avascular zone gradually decrease as kidney function decreases^16^. Superficial parafoveal density and superficial foveal density were shown to be lower in eyes of patients with than without systemic lupus erythematosus (SLE), with these densities being negatively correlated with SLE Disease Activity Index^17^. To date, however, conjunctival and retinal vascular densities have not been investigated in MI patients. The present study used OCTA technology to assess changes in retinal density and conjunctival vascular densities in patients with MI.

## Materials and methods

### Research subjects

The retrospective case–control study was conducted at the Department of Ophthalmology and Cardiology in Nanchang University from October 2022 to December 2022. This study recruited 16 patients (32 eyes) with MI, and 16 healthy individuals (32 eyes) from the ophthalmic research center at Nanchang University matched 1:1 by age and sex to the MI patients. Demographic and clinical characteristics, including gender, age and the results of vision and fundus examinations and OCTA were recorded (Table 1). All study subjects were examined by the same ophthalmologist. All subjects were informed of the experimental methods and possible risks and all provided written informed consent prior to the start of this study.

**Table 1:** Demographic characteristics and clinical findings of patients with MI and HCs.

Condition	HC (n = 16)	MI (n = 16)	t	p
Sex (male/female)	7/9	7/9	NA	NA
Age (years)	62.69 ± 0.70	63.31 ± 0.82	− 0.578	0.568
Best-corrected visual acuity (R)	0.96 ± 0.08	0.60 ± 0.30	4.687	0.02
Best-corrected visual acuity (L)	0.84 ± 0.15	0.63 ± 0.26	2.82	0.22
Intra-ocular pressure (R)	14.97 ± 1.52	14.61 ± 2.29	0.517	0.513
Intra-ocular pressure (L)	15.48 ± 1.85	14.54 ± 3.14	1.035	0.258
Systolic pressure (mmHg)	124.44 ± 5.74	122.19 ± 13.19	0.626	0.085
Diastolic pressure (mmHg)	82.94 ± 6.30	84.51 ± 6.46	− 0.693	0.589

*HC* healthy control, *MI* myocardial infarction, *R* Right, *L* Left.

### Inclusion criteria

Patients in the MI group were required to meet the diagnostic criteria for MI diagnostic criteria, to be aged ≥ 18 years of age. Subjects in the control group were required to have no history of eye disease or eye surgery, to not wear contact lenses or corneal contact lenses during the two weeks prior to study enrollment, to have a refractive error of < 6.00D and intraocular pressure (IOP) of 10–21 mmHg in each eye, and to have no systemic immune systemic disease.

### Exclusion criteria

Individuals were excluded from this study if they had a systemic autoimmune disease such as Sjögren syndrome (SjS); had undergone ocular surgery within 6 months prior to study enrollment; had eye diseases, such as glaucoma, cataract, or vitreous opacities; had diabetes or another circulatory illness that could affect the eyes; were long-term smokers; or had contraindications for dilated pupils.

### Ethical considerations

The Ethics Committee of The First Affiliated Hospital of Nanchang University (Jiangxi Province, China) approved this study, and all of the methods applied in it adhered to the goals of the Helsinki Declaration (as updated in 2013).

### OCTA imaging

OCTA imaging was performed by the same physician utilizing the RTVue Avanti XR system (Optovue, Fremont, CA) to see the microvasculature and retinal cross section simultaneously. In this study, we used a scanning speed of 70,000 wavelengths per second, a central wavelength of 840 nm, and a scan with a bandwidth of 45 nm to achieve the best imaging results. At the same time, we set the axial resolution to 5 mm and the horizontal resolution to 22 µm. During the angiogram, we used a pattern of 3 × 3 mm scans along the X-axis and performed 5 repeat scans to image at 216 grating positions along the Y-axis. The acquisition duration for each scan was 3.9 s, with the majority of the scans concentrated in the fovea.

Using two horizontal and two vertical gratings, we performed a series of four volume scans to obtain 3 × 3-mm OCTA images. Three-dimensional 3 × 3-mm en-face OCT angiograms were computed for each eye. Some studies have described that orthogonal scan alignment algorithm was used to correct motion artifacts. The OCTA images were produced using RTVue XR and the vessels were divided using the SSADA algorithm. It computes the link with the speckle amplitude at a given pixel and position. The relationship between speckle amplitude may be related to the pulsating motion of blood vessel. By lowering noise from axial motion, the RTVue XR Avanti system increases the detection signal-to-noise ratio through the use of OCT at four distinct frequency bandwidths. Four sets of voxel data have been collected at the same resolution since to this advancement. Pixel decorrelation was conducted at each frequency bandwidth in order to determine the average signal. Background, tissue motion, and blood flow sounds are reduced using high-resolution correlation. The signal of decorrelation lessens the anomalies^18^.

**Figure 1:**
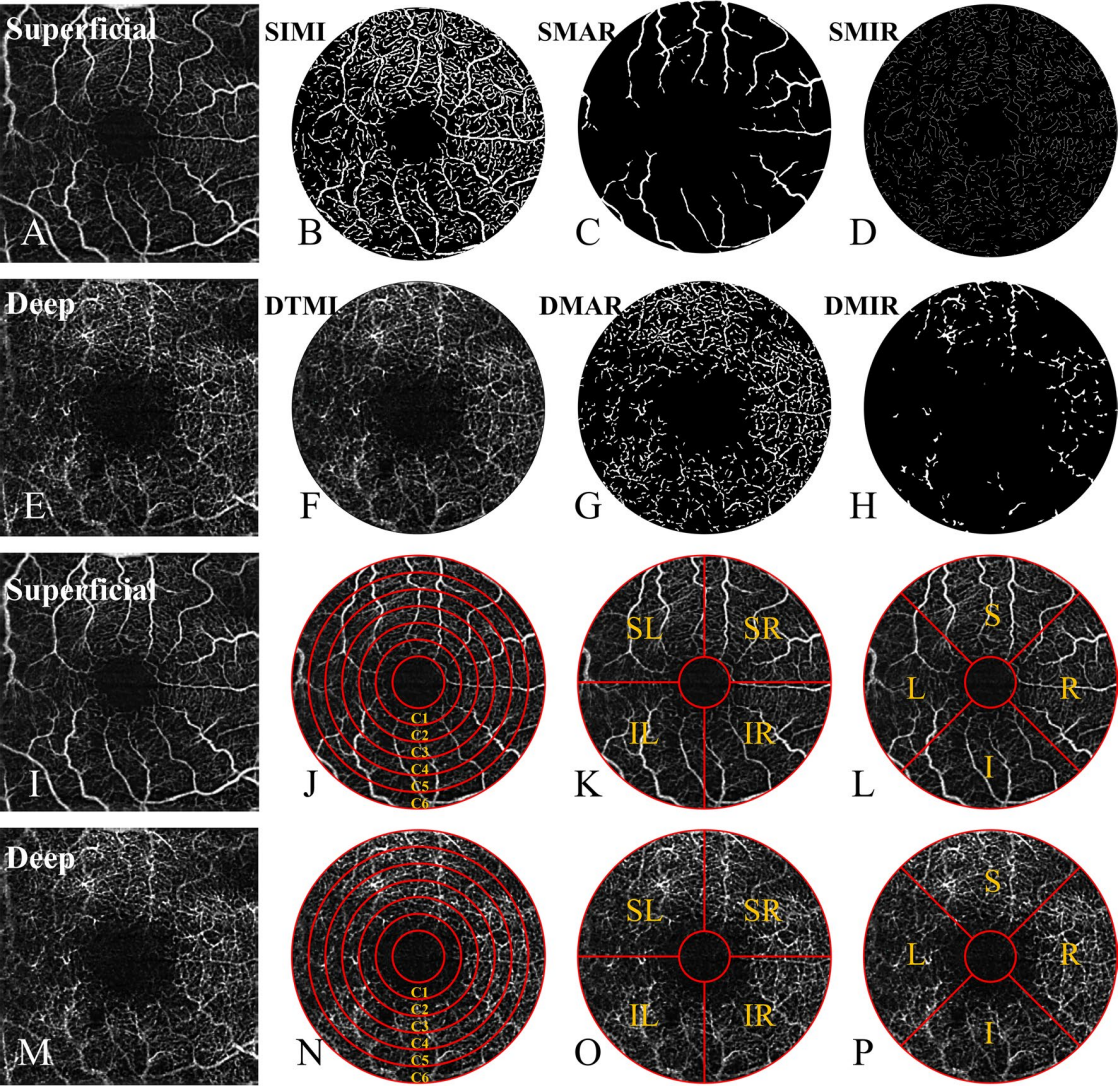
The 3 × 3-mm optical coherence tomography angiography image of the macular region of the retina (**A**–**H**). *STMI* Superficial total microvascular, *SMAR* Superficial macrovascular, *SMIR* Superficial microvascular, *DTMI* Deeper total microvascular, *DMAR* Deeper macrovascular, *DMIR* Deep microvascular. Partition methods of the retinal microvascular (**I**–**P**). *R* right, *L* left, *S* superior, *I* inferior, *SR* superior right, *SL* superior left, *IR* inferior right, *IL* inferior left.

For a more detailed analysis, we divided the retina into superficial and deep retina. We focused on MIR (Fig. 1D,H), MAR (Fig. 1C,G) and TMI (Fig. 1B,F) respectively for both superficial retinal layer (SRL) (Fig. 1A–D,I–L) and deep retinal layer (DRL) (Fig. 1E–H,M–P), and used fractal dimension to study the specific morphology and structure of fundus microvascular. Retinal vascular density is defined as the percentage of vascular perfusion area to the measured area. When calculating blood vessel density, we captured a 1080 b-scan at 270 frames per second at 216 y positions × 5 positions^19^.We created a two-dimensional front image of the superficial retina using a threshold algorithm, and estimated the image blocks and assign each pixel as perfusion (1) or background (0).The vessel density has been measured using a comparable length-based metric. Using the mean of the skeletonized slab within an area of interest as the foundation, the vessel density was computed and scaled according to the pixel distances (512 pixels per 3 mm). Next, we calculated an image of blood vessel density from the fovea of the macula to the edge of the image, scaled according to pixel distance. After that, we processed the image with Bernoit software using a number of unique segmentation techniques. These algorithms include inversion, balancing, removal of non-vascular structures, and background noise processing to produce binary images. Through the application of these algorithms, we can effectively process the image, improve the clarity and easy analysis of the image. Finally, a 3 mm*3 mm macular area is created from the skeletonized superficial/deep complete microvascular picture.

**Figure 2:**
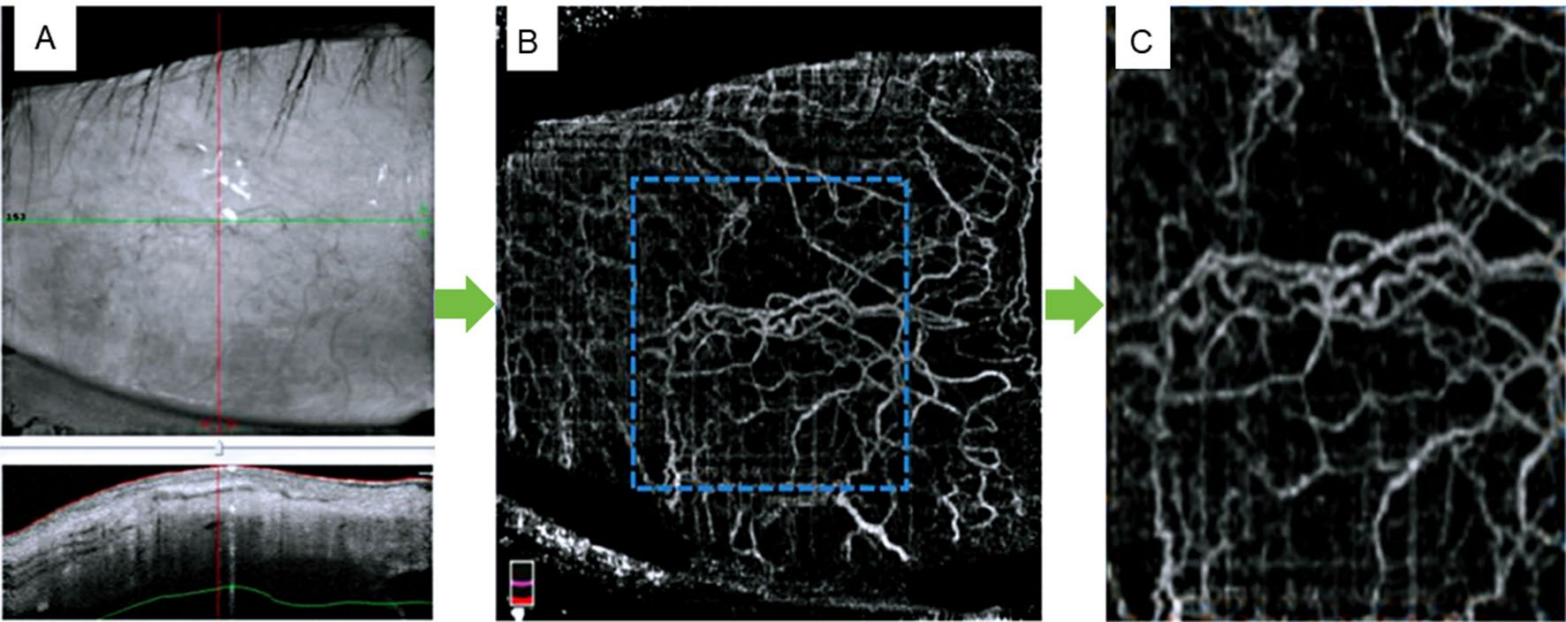
The optical coherence tomography angiography image of the microvascular of the conjunctiva. (**A**) Images of the patient’s conjunctiva. (**B**) OCTA scan of the conjunctiva with microvascular density. (**C**) Microvascular density of 3 × 3 mm^2^ in region of interest.

Conjunctival microvascular images were acquired using the same OCTA with the same parameters as before (Fig. 2). The lens adapter was set 2–4 cm from the corneal surface of the subject and was used to make pre-ventilated contact between the adapter lens and the eye, manually adjust the focus and focus until the image was clear. The limbus conjunctivae edge and 5 successive rectangles measuring about 4 mm each were determined to be the scanned areas. Microvascular structures were highlighted by selecting filters to generate binary images for analysis.

### Macular retinal segmentation method

(1) The annular partition method: the fovea vascularless region with a diameter of 0.6 mm was removed and the annular region with a diameter of 0.6–2.5 mm was subdivided into six equally wide thin rings with a bandwidth of 0.16 mm, which were named C1–C6.

(2) The hemispheric segmentation method: the macular retina was divided into four equal parts using vertical and horizontal lines, named as the superior right (SR), inferior right (IR), superior left (SL), and inferior left (IL).

(3) The modified early treatment of diabetic retinopathy study (ETDRS) method: by connecting two quadrant of the diagonal, the retina is divided into four quadrants, including superior (S), inferior (I), right (R) and left (L)^20^.

### Statistical analysis

Statistical analyses were performed using IBM SPSS Statistical software, version 27.00, and graphs were plotted using GraphPad Prism 9.5.1 software. Quantitative data were expressed as mean ± standard deviation (SD) and rank data were expressed as absolute number. Quantitative data were compared in the MI and healthy control groups by independent sample t tests, with p values < 0.05 regarded as statistically significant. The standard difference between the means of the two groups was displayed using Cohen's d effect size in the T-test. Generally speaking, Cohen's d values of 0.2–0.5 indicate small effects, 0.5–0.8 indicate medium effects, and 0.8 and higher indicate large effects. Receiver operating characteristic (ROC) curves were plotted, and the area under the curve (AUC) and critical point of each parameter were determined to assess the superficial and deep retinal blood flow density in the two groups. Correlations between retinal vascular density in the macular region and conjunctival blood flow density were determined by Pearson correlation analysis with the test level α = 0.05.

## Results

### Superficial macular vascular density

**Figure 3:**
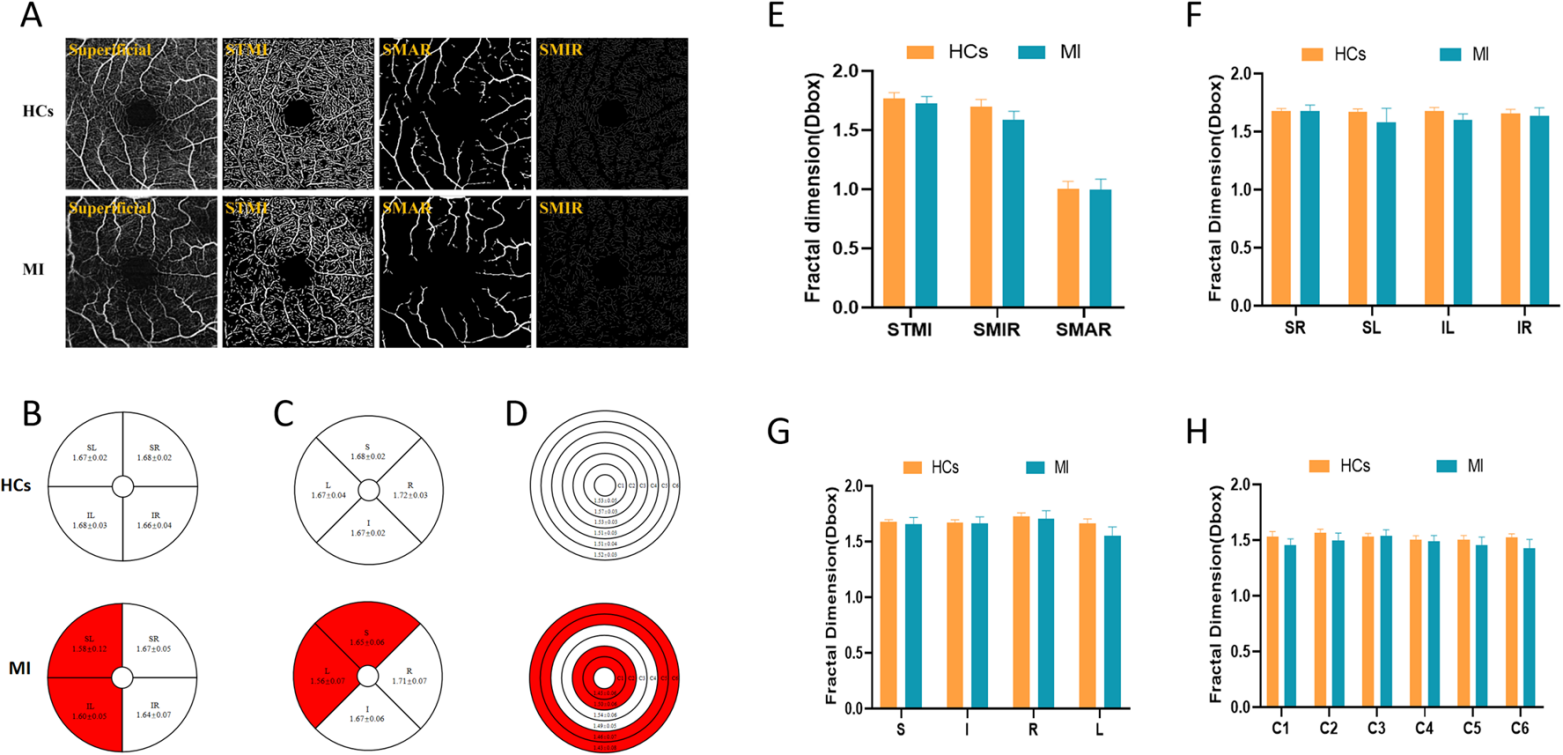
Comparison of superficial vessel density in different retinal regions between MI and control groups. (**A**) Superficial retinal vessel density map. (**B**) Hemisphere segmentation method in superficial retina layer. (**C**) The modified early treatment of diabetic retinopathy study (ETDRS) method in superficial retina layer. (**D**) The annular partition method in superficial retina layer. (**E–H**) Histogram of superficial vascular density in normal and MI groups. *STMI* superficial total microvascular, *SMIR* superficial microvascular, *SMAR* superficial macrovascular, *R* right, *L* left, *S* superior, *I* inferior, *SR* superior right, *SL* superior left, *IR* inferior right, *IL* inferior left.

Evaluation of the TMI, MAR, and MIR densities in the SRL (Fig. 3A) showed that STMI (P < 0.001, Cohen's d = 2.24), SMIR (P = 0.001, Cohen's d = 0.86), and SMAR (P < 0.001, Cohen's d = 0.94) retinal densities in the superficial macular area were significantly lower in the MI than in the control group (Fig. 3E). Comparisons of microvascular density by the hemispheric segmentation method showed that vessel densities in the SL (P < 0.001, Cohen's d = 1.08) and IL (P < 0.001, Cohen's d = 1.78) regions of the SRL were significantly lower than in the MI than in the control group (Fig. 3B,G). The modified ETDRS segmentation method showed that vessel densities in the S (P = 0.031, Cohen's d = 0.56) and L (P < 0.001, Cohen's d = 1.85) regions were also significantly lower in the MI than in the healthy control group (Fig. 3C,H). Similarly, the annular partition method showed that vessel densities in the C1 (P < 0.001, Cohen's d = 1.42), C2 (P < 0.001, Cohen's d = 1.37), C5 (P < 0.001, Cohen's d = 0.88), and C6 (P < 0.001, Cohen's d = 1.57) regions were significantly lower in the MI group than in the control group (Fig. 3D,F). Vessel densities in other regions, however, did not differ significantly in the two groups (Fig. 3).

### Deep macular vascular density

**Figure 4:**
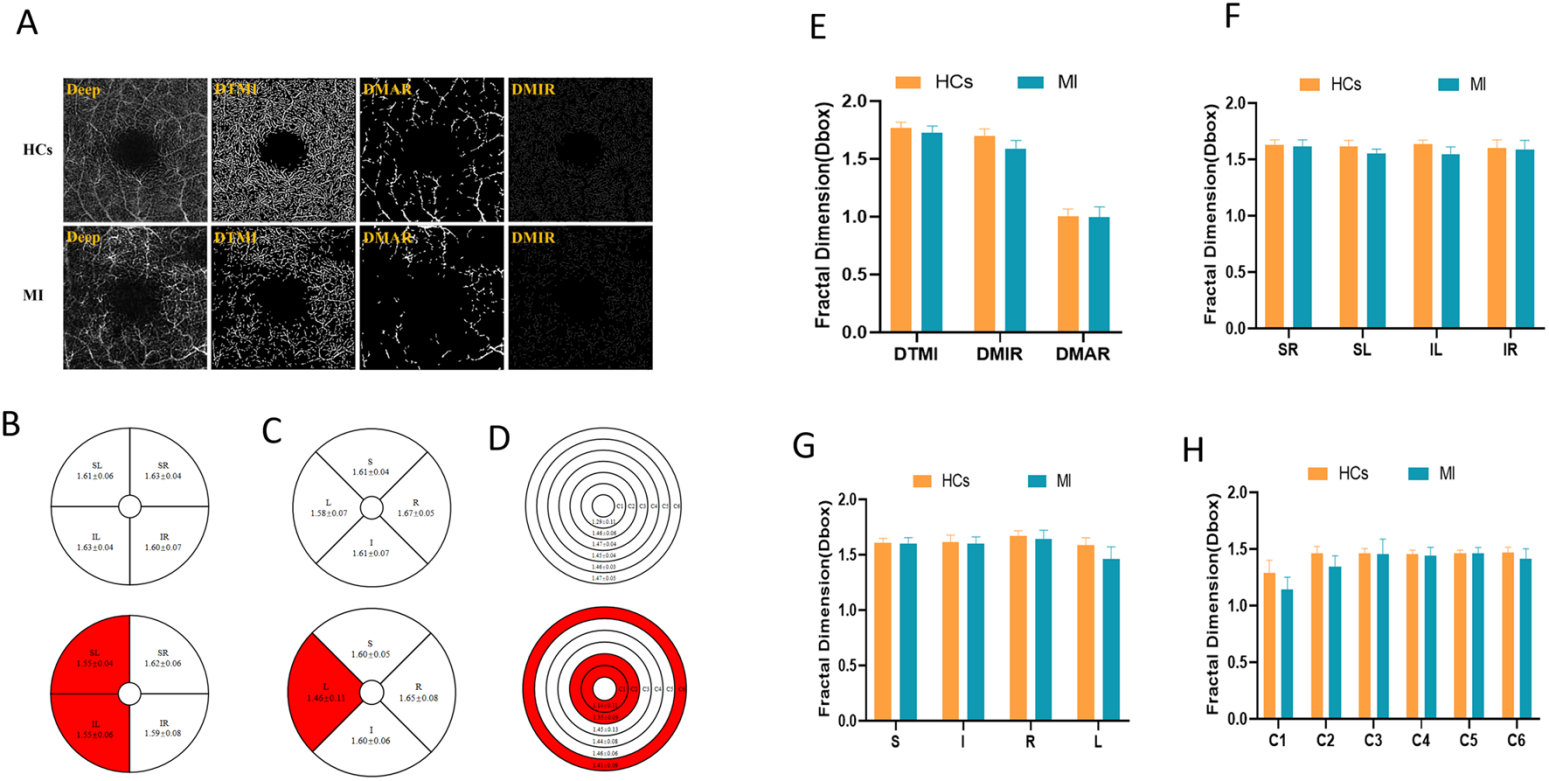
Comparison of deep vessel density in different retinal regions between MI and control groups. (**A**) Deep retinal vessel density map. (**B**) Hemisphere segmentation method in superficial retina layer. (**C**) The modified early treatment of diabetic retinopathy study (ETDRS) method in superficial retina layer. (**D**) The annular partition method in superficial retina layer. (**E**–**H**) Histogram of deep vascular density in normal and MI groups. *DTMI* Deeper total microvascular, *DMAR* Deeper macrovascular, *DMIR* Deep microvascular, *R* right, *L* left, *S* superior, *I* inferior, *SR* superior right, *SL* superior left, *IR* inferior right, *IL* inferior left.

Comparisons of the TMI, MAR and MIR densities in the DRL (Fig. 4A) showed that the DTMI (P = 0.003, Cohen's d = 0.78) and DMIR (P < 0.001, Cohen's d = 1.63) densities were significantly lower in the MI than in the control group, whereas their DMAR densities did not differ significantly (Fig. 4E). The hemispherical segmentation method showed that blood vessel densities in the SL (P < 0.001, Cohen's d = 1.22) and IL (P < 0.001, Cohen's d = 1.69) regions were significantly lower in the MI than in the control group (Fig. 4B,G), and the modified ETDRS segmentation method showed that microvascular density in the L (P < 0.001, Cohen's d = 1.31) region was significantly lower in the MI than in the control group (Fig. 4C,H). The ring segmentation method showed that vessel densities in the C1 (P < 0.001, Cohen's d = 1.36), C2 (P < 0.001, Cohen's d = 1.49) and C6 (P = 0.002, Cohen's d = 0.82) regions were significantly lower in the MI than in the control group (Fig. 4D,F).

### Conjunctival blood vessel densities

**Figure 5:**
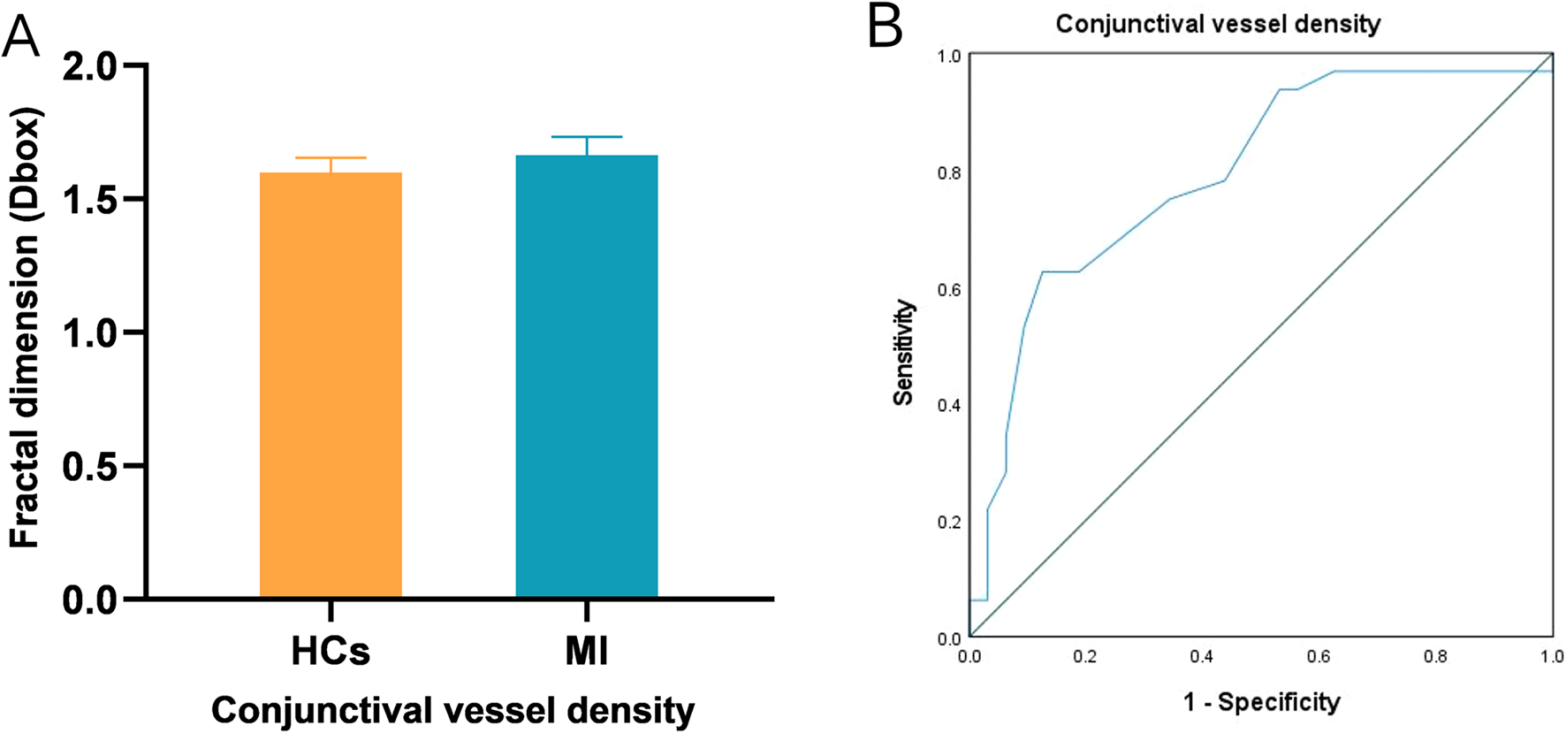
Conjunctival vascular density evaluation in the healthy control and MI groups. (**A**) Statistic analysis of conjunctival vascular density in two groups; (**B**) ROC curve analysis of conjunctival microvascular density between two groups.

OCTA showed that the vessel density of the temporal conjunctiva was significantly higher in the MI than in the healthy control group (P < 0.001, Cohen's d = 1.04, Fig. 5).

### ROC analysis of retinal (superficial and deep) and conjunctival vessel densities

**Figure 6:**
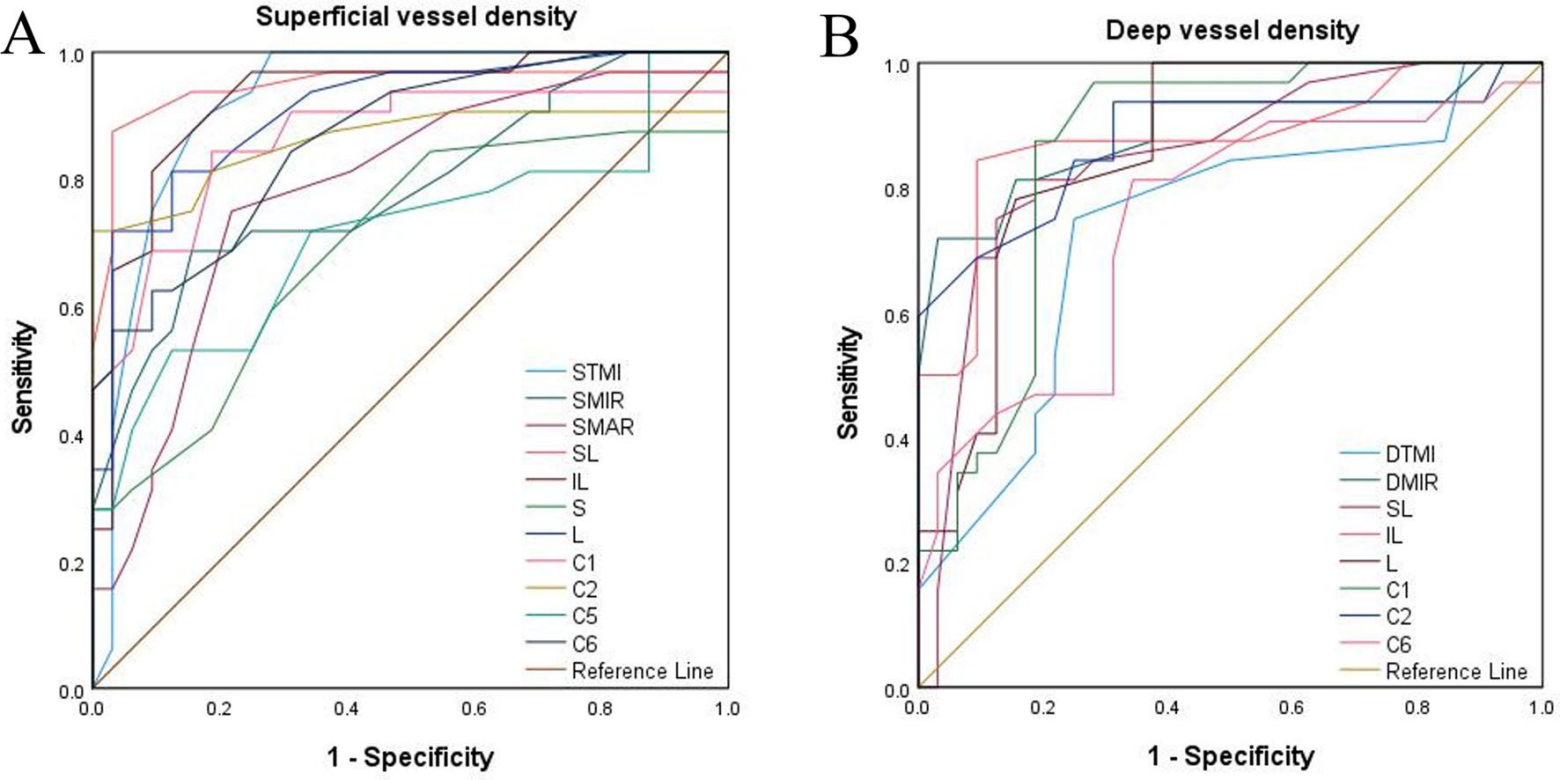
(**A**) ROC curve analysis of quadrantal, sectorial, and annular microvessel densities in the superficial layers. (**B**) ROC curve analysis of quadrantal, sectorial, and annular microvessel densities in the deep layers. *STMI* Superficial total microvascular, *SMAR* Superficial macrovascular, *SMIR* Superficial microvascular, *DTMI* Deeper total microvascular, *DMAR* Deeper macrovascular, *DMIR* Deep microvascular. Partition methods of the retinal microvascular. *R* right, *L* left, *S* superior, *I* inferior, *SR* superior right, *SL* superior left, *IR* inferior right, *IL* inferior left.

OCTA showed excellent specificity and sensitivity in detecting retinal density variations in the MI and control groups. The STMI, SMIR, SMAR, SL, IL, S, L, C1, C2, C5 and C6 areas in the superficial retinal layer could be distinguished in the MI group (P < 0.05, Fig. 6A). The area under the ROC curve of superficial retinal density in the SL region was 0.945 (95% confidence interval [CI] 0.879–1.00), indicating that this region had high diagnostic sensitivity for MI. In the deep retinal layer, significant differences between the MI and control groups were detected in the DMIR, DTMI, SL, IL, L, C1, C2 and C6 regions (P < 0.05, Fig. 6B). The area under the ROC curve for MIR was 0.888 (95% CI 0.802–0.974), suggesting that DMIR has moderate diagnostic sensitivity for MI. The ROC curve of the conjunctival vessel density showed a significant difference between the two groups, with an area under the ROC curve of 0.792 (95% CI 0.681–0.904).

### Relationship between macular and conjunctival vascular density

Analysis of the relationships between the densities of the vascular vessels in the conjunctiva and retina showed no correlations in either the MI or healthy control group.

## Discussion

To our knowledge, this is the first published study on OCTA measurements of ocular microvascular changes in MI patients. The study found that both microvascular and macrovascular densities were lower in MI patients than in normal controls.

MI has been defined as a sudden reduction or cessation of blood flow through the coronary arteries, resulting in the ischemic necrosis of corresponding myocardial cells. MI is the most prevalent severe cardiologic condition and a major contributor to heart failure. The mortality rate from MI has increased over the past few years^21^. MI occurs when plaque, cholesterol, and fat deposits narrow or block the coronary arteries, leading to blood clots that reduce blood flow to the heart. Atherosclerosis as a major risk factor for MI, significantly affecting both the main heart vessels and the microcirculation, including the retinal and choroid vascular systems, leading to vascular damage and blood flow restriction through the sparse capillary network^22^.

Microvascular anomalies are increasingly recognized as important components of MI. Despite increased interest in the contribution of microvascular damage to MI, the methods currently available to directly depict microcirculation in the heart have some limitations, such as being invasive, time-consuming, operator dependent, technically difficult, expensive, and not widely accessible. Examination of the retinal microcirculation may provide an alternative. OCTA has made possible the detailed evaluation of retinal structure and microcirculatory function. Cardiovascular conditions have been associated with alterations in the ocular micro-vessels^23^. For example, variations in retinal thickness and alterations in the characteristics of the retinal microvasculature were found to be associated with cardiovascular health^8,10^. Hypertension was found to be significantly associated with an increase in foveal avascular area and a decrease in blood vessel density, changes that may be due to the chronic effects of high blood pressure caused by the microcirculation, resulting in ischemia and reduced retinal thickness^24^.

The present study utilized OCTA to compare the retinal vascular density of MI patients with that of healthy controls. Software was used to divide the retina into two layers, the superficial and deep retinal layers, with the retina also segmented using several types of segmentation methods. The density of the superficial capillaries in the STMI, SMIR, SMAR, SL, IL, S, L, C1, C2, C5 and C6 regions was found to be significantly lower in MI patients than in healthy controls (P < 0.05 each). In addition, the density of deep capillaries in the DMIR, DTMI, SL, IL, L, C1, C2 and C6 regions was found to be significantly lower in MI patients than in healthy control individuals.

The present findings, showing that low superficial and deep retinal layer capillary plexus vascular density was associated with MI, were in agreement with previous findings. Cardiovascular disorders in general may be associated with lower vessel density, suggesting that retinal microvascular abnormalities may be associated with cardiovascular risk profiles. The density of Superficial capillary plexus (SCP) vascular density was found to be significantly lower in children with than without chronic heart failure^25^. In addition, vessel density throughout the entire retina, including both the superficial and deep plexuses, was found to be significantly lower in patients with than without coronary heart disease (p < 0.001)^26^. Furthermore, results from the EYE-Myocardial Infarction study showed that VD in SP was lower in patients with than without acute coronary syndrome^27^. Microvascular networks in different layers of the retina may act as surrogate indicators of microvascular function, with retinal microcirculatory damage being an indicator of systemic macrovascular disorders^28^.

The retina is composed of ten different anatomical layers and supplied with blood by two sources^29,30^. Fundus blood vessels are divided into those of the inner retina (divided into superficial and deep capillary layers), the outer retina and the choroidal vascular plexus. The inner retinal vascular system is located in the ganglion cell layer, whereas the outer retinal vascular system is located in the core layer^31^. OCTA has confirmed that the retina has two sets of relatively independent blood vessel networks, one in the superficial layer and the other in the deep layer, with vertical blood vessels providing communications between these networks^32^. The SCP is a network of blood vessels that is joined to the retinal arteries and veins. The SCP consists of both bigger and smaller blood vessels that supply downstream capillary clusters. The contraction and relaxation of smooth muscle and endothelial cells in the retinal arteries and arterioles is the primary mechanism through which blood perfusion in SCP is self-regulated. The deep capillary plexus (DCP) consists of finer and more uniform retinal capillaries, without larger blood vessels connecting the capillary plexus, with each capillary unit consisting of a continuous layer of endothelial cells surrounded by pericytes^33^. The structure and function of the retina are complex, resulting in a high demand for oxygen, correctly regulated hemodynamics and delivery of metabolic substrates^34^. Vascular diseases result in mechanical damage, deterioration of the efficiency of the endothelial barrier, and impaired capillary function. Neural activity and energy production in the retina result in the release of nitric oxide, lactate, and arachidonic acid metabolites by neurons and glial cells, with interactions of these metabolites optimizing blood flow. During the development of microvascular disease, the interactions of these metabolic processes are altered by impairments in retinal neuronal tissue and endothelium, resulting in a disordered control of blood flow^35^. Furthermore, retinal endothelial cells are more vulnerable to oxidative stress, due to an imbalance between superoxide and superoxide dismutase in these cells. Because the development of MI involves reactive oxygen species (ROS)^36^, MI has an impact on the retinal endothelium. To maintain the autoregulation of blood flow in the retina, retinal arterioles adapt to changes in perfusion pressure (pressure autoregulation) and metabolic demands (metabolic autoregulation)^37,38^. Anatomically, the superficial capillary plexus consists of smooth muscle cells, whereas the deep capillary plexus contains capillaries composed of endothelial cells, basement membrane, and pericytes^39^. Because pericytes are more susceptible to hypoxia, the deep capillaries are more susceptible to ischemia and circulatory abnormalities than the superficial capillaries^40^.

Cardiovascular risk may also be associated with retinal microvascular abnormalities. This may result in the development of the early phases of epicardial and non-endothelium-dependent microangiopathy, resulting in the gradual depletion of the coronary reserve^41^. Many risk factors for MI, such as smoking, hypertension, and dyslipidemia, are also major precursors of microvascular abnormalities. Although retinal microvascular abnormalities have been associated with cardiovascular diseases, the underlying pathogenesis of the link between small and large vascular diseases remains poorly understood. After adjusting for other risk factors, retinal arteriolar stenosis was found to be an independent predictor of new coronary heart disease, suggesting that damage to the retinal arterioles may result from microvascular damage induced by conditions such as diabetes, intimal thickening, and excessive blood pressure^42^. A study assessing the associations between retinal vascular diameter and several cardiovascular risk factors found that both higher homocysteine concentrations and hypertension correlated with lower retinal arteriolar diameter. Diabetes, smoking, obesity, dyslipidemia, and inflammatory indicators, including C-reactive protein and fibrinogen, were all linked to larger venules^43^. Evaluation by static fundus color photography of retinal artery changes in cardiovascular disease patients with multiple risk factors found that, after adjusting for these risk factors, the structure and function of the retinal microvessels were strongly correlated with endothelial dysfunction^44^. Endothelial malfunction is the initial vascular damage caused by inflammation and oxidative stress^45^. Age, hypertension, diabetes, smoking, obesity, dyslipidemia, and other cardiovascular disease risk factors can harm vascular endothelial cells, leading to vascular endothelial dysfunction and microvascular injury, including structural abnormalities like reduced vascular density and limited microvascular constriction and dilation. Both pericytes and endothelial cells have contractile characteristics that control blood flow in the capillary plexus^43^. Endothelial function also affects the occurrence and development of systemic arteriosclerosis^46^. Endothelial dysfunction may result in poor capillary blood flow control, which ultimately causes microcirculation ischemia, resulting in microvascular thinning, with OCTA showing reduced blood vessel density. These alterations all indicate microvascular diseases.

The conjunctival microcirculation can also be evaluated in clinical settings. The conjunctiva is the only part of the body surface where the entire microcirculatory process can be observed. The bulbo-conjunctival microcirculation has been reported to be altered in patients with diabetes^47^, hypertension^48^, and systemic lupus erythematosus^49^, suggesting that evaluation of the microcirculation may be diagnostic of these diseases.

The present study found that the density of the conjunctival microvasculature was considerably higher in MI patients than in normal controls. Increased spontaneous VEGF-A production following acute myocardial ischemia has been reported to induce compensatory angiogenesis by stimulating the ROS stress autophagy axis in vascular endothelial cells^50^.

The present study had several limitations. First, patients with MI were not categorized based on electrocardiographic alterations. In addition, this study included small numbers of MI patients and normal controls. Further research in larger cohorts of MI patients is required to determine the associations between MI and the retinal microvasculature, as well as the clinical applications of OCTA in these patients.

## Conclusions

The present study used OCTA to assess ocular microvascular alterations in patients with MI. Conjunctival microvascular density was found to be higher and macular microvascular density (in both the superficial and deep retinal layers) was found to be lower in MI patients than in normal controls. These alterations in microvascular densities indicate that OCTA is a promising technique for distinguishing MI-affected from healthy eyes. OCTA is a rapid, quantitative, noninvasive, and easily performed method, suggesting that it may be helpful in clinical practice for MI patients.

## Data Availability

The datasets used and/or analyzed during the present study are available from the corresponding author on reasonable request.
